# Why do the impacts of coronavirus disease 2019 and the response surprise the world?

**DOI:** 10.4102/jamba.v12i1.1028

**Published:** 2020-12-09

**Authors:** Emmanuel Raju, Dewald van Niekerk

**Affiliations:** 1 Unit for Environmental Sciences and Management, African Centre for Disaster Studies, North-West University, Potchefstroom, South Africa; 2 Global Health Section and The Copenhagen Centre for Disaster Research (COPE), University of Copenhagen, Copenhagen, Denmark

If disasters can be used as a prism to study inequality in terms of who gets affected the most, the coronavirus disease 2019 (COVID-19) pandemic has yet again revealed that society’s most vulnerable are worst affected. Whilst governments and the myriad range of actors are finding novel ways to contain the virus, lessons from previous disasters might serve as a useful strategy in designing a more humane response. Every corner of the world has now heard of and/or imposed ‘lockdowns’ to contain the virus. However, in low- and middle-income countries, what impact does the lockdown bring and on whom? Hasty decisions to close borders without notice and impose lockdowns have left millions stranded, out of work and are even pushing people into poverty (or further into poverty). The pandemic has entered some of the most vulnerable settings, such as informal settlements and refugee camps (Raju & Ayeb-Karlsson 2020). Disaster research has for decades highlighted components of poverty, vulnerability, structural inequality and marginalisation as crucial factors in the making of disasters. Yet, why does this come as a surprise? We highlight the need for more engagement with disaster scholars in addressing this global pandemic.

Disasters are not natural, but hazards are (Hewitt 1983). The social science of disaster research has time and again for over 40 years emphasised the need to move away completely from using the term ‘natural disasters’. However, at the Global Platform (GP) for Disaster Risk Reduction, held in Geneva in May 2019, there were many institutions using the phrase without a second thought or reflection. This use of the term ‘natural disasters’ takes away the accountability of states and other responsible actors to avoid risk creation. In a health context, for example, this extends to looking at why are vulnerable populations worst affected or affected differently and how can everyone have access to healthcare in such a setting. Taking an example from a different disaster, during the post-earthquake (2015) zones of Nepal in February 2019, it became evident how difficult it is to reach many areas, given the difficult terrain and landscape of the landlocked country (Pyda et al. 2019; Rolsted & Raju 2020). Health is not a stand-alone sector and access to healthcare is influenced by many factors that arise from sociopolitical, economic and cultural dimensions. These enablers of access to healthcare need to be discussed during disaster preparedness, and not only in the aftermath of disasters. The direct relationship between disasters and health is established and needs no further elaboration, as is seen with disaster mortality and other indicators (Schmid & Raju 2020). However, the less spoken-about aspects of vulnerability and its determinants, including health variables, need a detailed investigation that would enable policymakers to push for change in this regard. The Sendai Framework for Disaster Risk Reduction (UNISDR 2015) places importance on health resilience and the World Health Organization released the Health Emergency and Disaster Risk Management Framework at the GP. This is also a call for more research to elaborate on the understudied aspects of health and DRR before a disaster, and not focus primarily on disaster response and health (Chan & Murray 2017).

Health has received little attention within disaster risk management and risk reduction. The start of 2020 with the severe acute respiratory syndrome coronavirus 2 (SARS-CoV-2) in Wuhan, China, sent the message again for more alignment between different global strategies and local interventions. The pandemic spread at a rate that the entire world has been affected (Huang et al. 2020). This requires countries to be more alert, not only at the start of identifying cases, but to ensure that health systems are able to respond and cope with this. In this regard, it must be remembered that not everyone is affected the same way. Disaster research has shown for decades that people living on the margins of society suffer most during disasters and hazardous impacts. During the ongoing pandemic, studies highlighted that we see disproportionate impacts amongst ethnic minorities, and social determinants of health must be factored in seriously (Khunti et al. 2020). This is also true with regard to informal settlements, slums and migrant workers as they seem to be left out of the mainstream COVID-19 response (Raju & Ayeb-Karlsoon 2020; Wilkinson 2020). A secondary impact of the pandemic will be the economic hardship that millions of people will experience. These impacts will also be on the most marginalised people.

Disaster risk reduction (DRR) needs to be seen in the light of addressing complex systems and therefore should strive for more adaptive systems (Coetzee, Van Niekerk & Raju 2016). Vulnerability must be at the heart of these decisions. This also means to stop the blame game of coordination and collaboration and move towards a truly coherent and integrated approach to address health in DRR. The recent article by Pyda et al. (2019) accounts for important progress in the field of surgical planning and disaster preparedness, which could serve a useful strategy in emergencies globally. The co-chair’s summary from the GP highlights that ‘[*p*]lanning and action to manage biological hazards, including epidemics and pandemics needs to be strengthened, whilst enhancing investments in resilient health facilities’ (UNDRR 2019a). However, for DRR to be taken seriously, we need to move away from ‘natural disasters’ as this would bring the focus into planning and rigorous systems.

The year 2015 was a crucial year for negotiations of different international frameworks. They are the Sendai Framework for Disaster Risk Reduction (UNISDR 2015), the Paris Agreement on Climate Change (2015) and the 2030 Sustainable Development Goals (SDGs). Of particular relevance are also the Grand Bargain at the World Humanitarian Summit (2016), the New Urban Agenda (2017) (United Nations 2017) and the Addis Ababa Action Agenda (2015) (United Nations 2015). These frameworks have many aspects in common and aim for a sustainable world. Amongst others, human health and well-being are one of the core components of all these frameworks (Aitsi-Selmi & Murray 2016; Chan & Murray 2017). The crucial question is whether health and human well-being will be prioritised beyond the COVID-19 response and considered sufficiently in overall DRR. Whilst the virus respects no national boundaries, sharing lessons from the past and best practices from the present is the utmost need of the hour.

The GP highlighted the absolute need for more engagement of health as a theme, and with more public health stakeholders in general (UNDRR 2019a). Coetzee et al. (2016) emphasised the need for systems with more holistic thinking in addressing complex adaptive issues, such as disaster risks and, in particular, extensive risk. The aim must be to devise robust, flexible and resilient adaptive systems that work in harmony with all sectors. Whilst there is an increasing amount of work in the context of health and DRR, sufficient efforts to recognise that much needs to be done about the health-development-disaster nexus are lacking. Furthermore, the upcoming Conference of the Parties (COP 26) must place human health and the impacts of climate change on health at the heart of all discussions. The Lancet report highlights that infectious diseases burden could worsen with climate change (Watts et al. 2016). This calls for more synergies between actors in public health institutions working on DRR and climate change adaptation.

Finding the correct response to a global pandemic remains an elusive question for most of those who are affected: ensuring that the marginalised and poor communities do not find themselves removed from various systems, but are a part of, and impacted on, by a myriad of systems (both positively and negatively). The complex (not complicated!) nature of integrated, nested and linked systems must be acknowledged and used to develop DRR solutions, which are multi-scalar, multi-dimensional and multi-sectoral in nature.
